# Macrophage-Associated Mesenchymal Stem Cells Assume an Activated, Migratory, Pro-Inflammatory Phenotype with Increased IL-6 and CXCL10 Secretion

**DOI:** 10.1371/journal.pone.0035036

**Published:** 2012-04-04

**Authors:** Kevin Anton, Debabrata Banerjee, John Glod

**Affiliations:** 1 Department of Pharmacology, The Cancer Institute of New Jersey, Robert Wood Johnson Medical School, University of Medicine and Dentistry of New Jersey, New Brunswick, New Jersey, United States of America; 2 Department of Medicine, The Cancer Institute of New Jersey, Robert Wood Johnson Medical School, University of Medicine and Dentistry of New Jersey, New Brunswick, New Jersey, United States of America; 3 Department of Pediatrics, The Cancer Institute of New Jersey, Robert Wood Johnson Medical School, University of Medicine and Dentistry of New Jersey, New Brunswick, New Jersey, United State of America; University of Sao Paulo – USP, Brazil

## Abstract

Mesenchymal stem cells (MSCs) exhibit tropism for sites of tissue injury and tumors. However, the influence of the microenvironment on MSC phenotype and localization remains incompletely characterized. In this study, we begin to define a macrophage-induced MSC phenotype. These MSCs secrete interleukin-6 (IL-6), CCL5, and interferon gamma-induced protein-10 (CXCL10) and exhibit increased mobility in response to multiple soluble factors produced by macrophages including IL-8, CCL2, and CCL5. The pro-migratory phenotype is dependent on activation of a c-Jun N-terminal kinase (JNK) pathway. This work begins to identify the influence of macrophages on MSC biology. These interactions are likely to play an important role in the tissue inflammatory response and may provide insight into the migratory potential of MSCs in inflammation and tissue injury.

## Introduction

It is well established that mesenchymal stem cells (MSCs) exhibit tropism for sites of tissue injury and the tumor microenvironment [1]. While the migratory ability of MSCs is well documented, the precise molecular mechanisms responsible for MSC homing to specific *in vivo* targets remain incompletely characterized. A better understanding of the MSC migratory process may identify therapeutic targets for the treatment of neoplastic and inflammatory disease and facilitate novel uses of MSCs such as targeted drug-delivery and gene therapy [2]–[4].

The inflammatory response plays a major role in forming the microenvironment of both injuries and tumors and the complex interplay between cellular components within this milieu influences the pathophysiology of these conditions. The microenvironment of tumor types such as breast cancer and high-grade gliomas is characterized by a dense population of macrophages [5]–[9]. Tumor-associated macrophages (TAMs) promote tumor progression by stimulating angiogenesis, inducing tumor cell invasion and metastasis, and conferring chemoresistant properties to tumor cells [10], [11]. Macrophages also play a pivotal role in normal wound healing and tissue repair [12], [13].

We tested the hypothesis that macrophages stimulate MSC localization to tumors and sites of injury and influence their molecular phenotype within the microenvironment. Our data show that macrophages, through the release of soluble factors, stimulate MSC motility and alter their cytokine secretion profile. Macrophages promote MSC migration through the production of soluble factors that activate the c-Jun NH2-terminal kinase (JNK) signaling pathway. A single cytokine or chemokine does not elicit the maximum MSC response. Our data suggest that the interaction between macrophages and MSCs impacts the inflammatory microenvironment.

**Figure 1:**
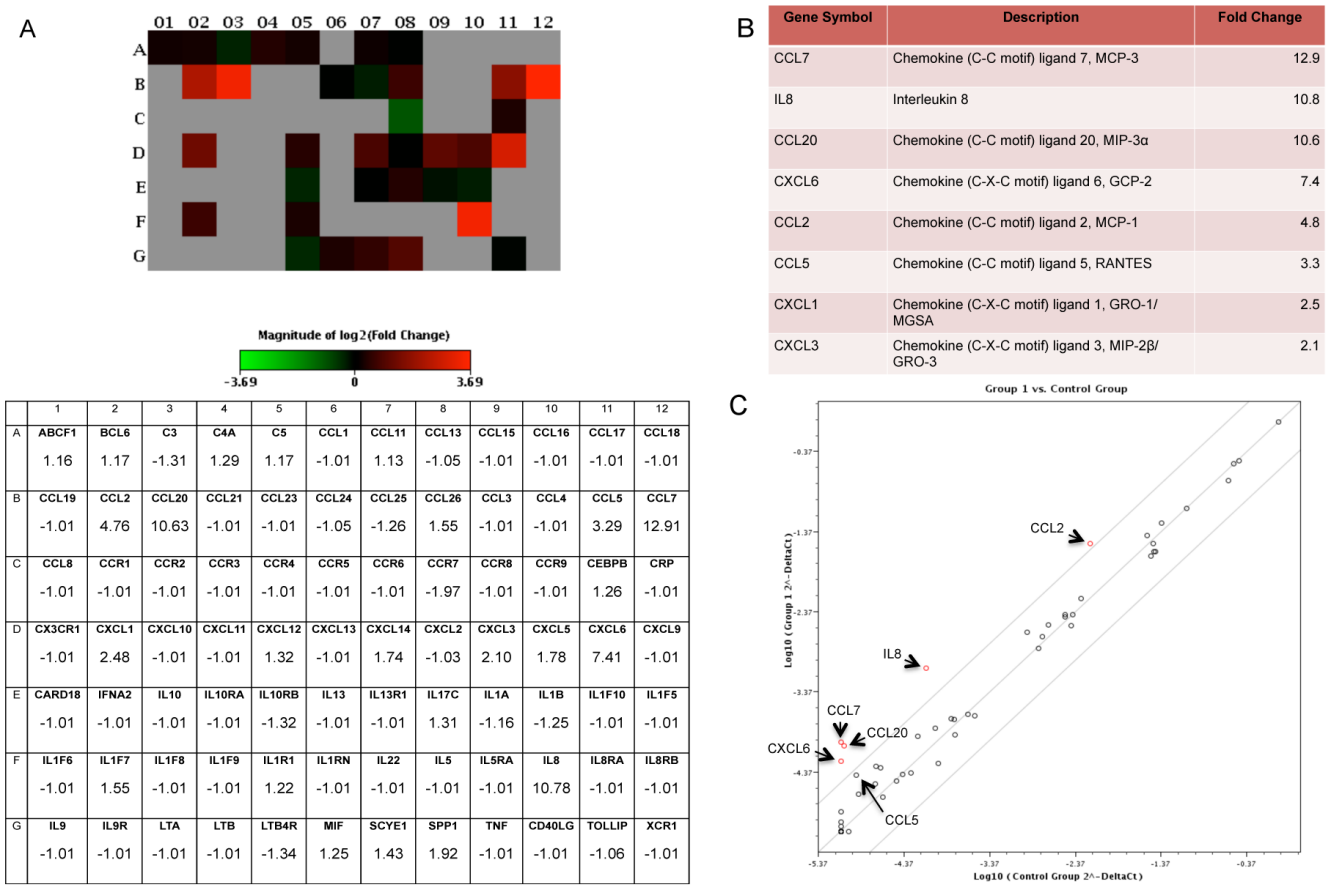
Inflammatory cytokine gene expression by MSCs is affected by macrophage-secreted soluble factors. MSCs exposed to macrophage CM for 24 hours express an altered inflammatory cytokine gene expression profile. Analysis of 84 key genes mediating the inflammatory response was conducted using RT-PCR generating a heat map expressing log-fold changes. Gray boxes represent genes that were undetected in either the control MSCs or the macrophage CM-stimulated MSCs (A). When stimulated by macrophage CM, MSCs increase the expression of inflammatory factors such as, CCL7, IL-8, CCL20, CXCL6, CCL2, CCL5, CXCL1, and CXCL3. The changes ranged from 2.1 to 12.9-fold (B). These data can also be visualized in a scatter plot, showing gene up-regulation as points scattered into the upper left quadrant (C).

## Results

### Macrophage-associated MSCs Up-regulate the Expression of Pro-inflammatory Cytokines

**Figure 2:**
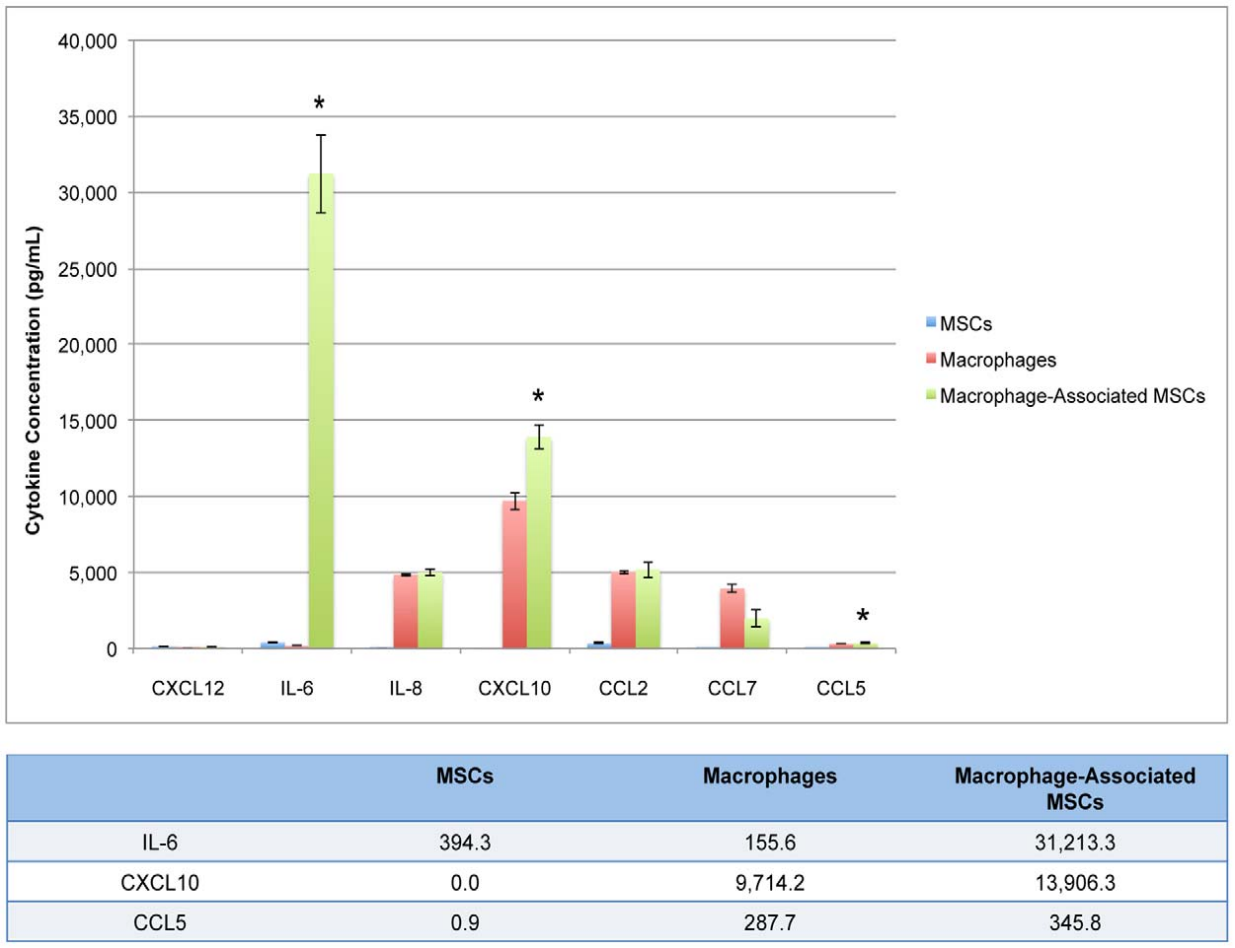
Macrophage-secreted soluble factors induce IL-6 and CXCL10 secretion by MSCs. Macrophages and MSCs have distinct cytokine secretion profiles that are variable and significantly dependent on the soluble factors and cells in their local environment. Both macrophages and MSCs are highly plastic and assume unique phenotypes when activated by paracrine factors. MSCs cultured in macrophage-conditioned medium for 24 hours assume a pro-inflammatory phenotype with increased secretion of IL-6 and CXCL10. Resting MSCs secrete 394 pg/mL and an undetectable amount of IL-6 and CXCL10, respectively. Upon activation by macrophage-conditioned medium, MSCs increased their secretion of IL-6 to 31,213 pg/mL and CXCL10 to 13,906 pg/mL. This represents a 56-fold increase in IL-6 (p<0.0001) and a 1.4-fold increase in CXCL10 (p<0.003). In addition, MSCs minimally increased the secretion of CCL5 by 1.2-fold from 287 pg/mL to 345 pg/mL (p <0.02).

Macrophages and MSCs are highly plastic cells that undergo changes in phenotype based upon local environmental cues. Both cell types are integral components of inflammation and are likely to interact during processes such as wound healing and solid tumor growth. In order to better define the impact of a macrophage-rich microenvironment on MSC phenotype we first identified changes in mRNA expression in response to culture in macrophage conditioned medium. MSCs exhibit changes in gene expression indicative of a pro-inflammatory phenotype, with increased expression of CCL7, IL-8, CCL20, CXCL6, CCL2 and CCL5 mRNA (Figure 1). We then determined whether increases in mRNA expression were accompanied by changes in cytokine secretion for five of the up-regulated genes in addition to IL-6, a cytokine that is highly expressed by pro-inflammatory MSC1 cells [14]. MSCs activated by macrophages had increased secretion of IL-6, CXCL10, and CCL5. To produce macrophage-activated MSCs, cell-free macrophage conditioned medium (CM) was harvested and added to MSC cultures for 24 hours. IL-6 levels in the medium increased from 394.3 pg/mL to 31,213.3 pg/mL and levels of CXCL10 and CCL5 were increased by 1.4- and 1.2-fold, respectively. Despite an increase in mRNA expression, IL-8 and CXCL12 did not show significant changes in secretion and CCL7 secretion was decreased despite a marked elevation in mRNA levels (Figure 2).

### Macrophages Secrete Soluble Factors that Stimulate MSC Migration

**Figure 3:**
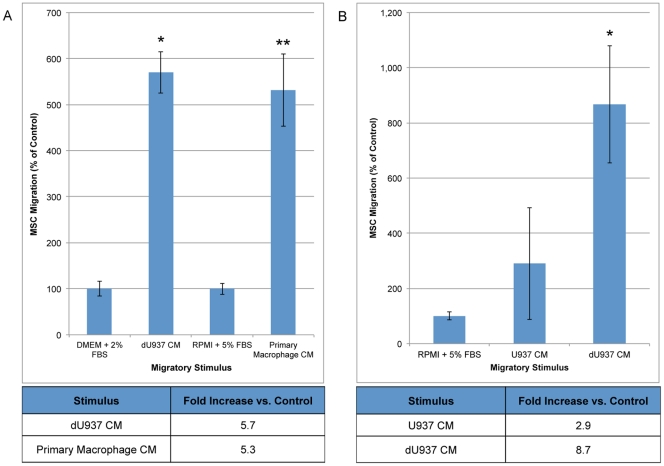
Macrophages induce MSC migration through the release of soluble factors. To determine the chemotactic potential of macrophages for MSCs the CM from primary culture macrophages and the dU937 macrophage cell line was harvested and used as the stimulus in a standard Boyden Chamber migration assay. Following an 18-hour migration, MSCs show increased motility and migration towards macrophage-like CM and primary macrophage CM when compared with control medium. Compared to control levels, the number of migrating MSCs is 5.7-fold greater towards dU937 CM (*p<0.003, n = 3) and 5.3-fold greater towards primary macrophage CM (**p<0.01, n = 3), respectively (A). A separate experiment was conducted to characterize the ability of dU937 macrophages alto induce MSC chemotaxis. When compared to control, the soluble factors secreted by the dU937 macrophage cell line induce an 8.7-fold increase in MSC migration while U937-secreted factors increased MSC migration 2.9-fold (B). dU937 macrophage cells induce a significantly higher amount of MSC migration than the undifferentiated cells (*p<0.03, n = 3).

We then investigated the ability of soluble factors produced by macrophages to induce MSC migration *in vitro*. Following an 18-hour migration, conditioned medium from both the human lymphoma cell line U937 differentiated to a macrophage phenotype (dU937) and primary culture human macrophages stimulated MSC migration (5.7- and 5.3-fold compared to control medium) (Figure 3A).

The ability of dU937 macrophages to induce MSC migration was compared with undifferentiated U937 cells (Figure 3B). Conditioned medium from U937 cells cultured without PMA (undifferentiated U937) stimulated a 2.9-fold increase in MSC migration compared to control medium. dU937 CM promoted a significantly higher level of MSC migration, inducing an 8.7-fold increase over control medium-stimulated MSCs.

### Macrophages Secrete an Array of Factors with the Capacity to Induce Migration of MSCs, Including CCL5, CCL2, and IL-8

**Figure 4:**
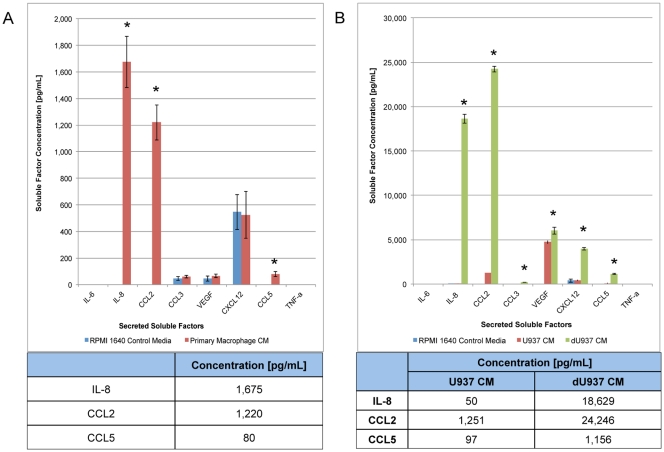
Identification of soluble factors produced by macrophages. Unstimulated macrophages generated from human peripheral blood monocytes secrete IL-8, CCL2, and CCL5 (A) and do not secrete detectable amounts of IL-6 or MIP-1α. dU937 macrophages secrete increased levels of IL-8, CCL2, CCL20, CCL5, VEGF, and CXCL12 when compared with both control medium and undifferentiated U937 CM (B). All samples were analyzed in triplicate. *p<0.05.

In order to identify factors involved in stimulating MSC chemotaxis, the cytokine profile of macrophage CM was determined. Macrophages secrete IL-8 (1,162 pg/mL), CCL2 (150 pg/mL), CCL5 (71 pg/mL), VEGF (260 pg/mL), and CXCL12 (643 pg/mL)(Figure 4A). Differentiated U937 cells displayed a cytokine profile with a similar pattern consisting of IL-8 (18,630 pg/mL), CCL2 (24,246 pg/mL), CCL3 (206 pg/mL), CCL5 (1,156 pg/mL), VEGF (6,011 pg/mL), and CXCL12 (3,970 pg/mL)(Figure 4B).

### CCL5, CCL2, and IL-8 are Chemotactic for MSCs

**Figure 5:**
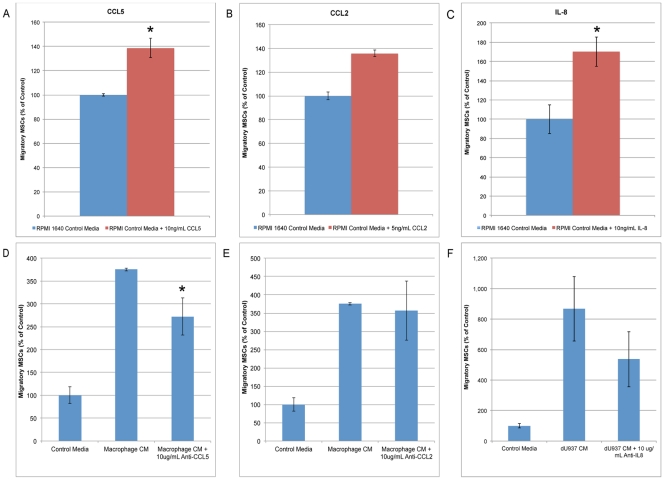
Stimulation of mesenchymal stem cells by CCL5, CCL2, and IL-8 secretion induces migration. To determine the role of macrophage-secreted CCL5, CCL2 (MCP-1), and IL-8 in MSC migration, the level of migration in response to recombinant proteins was analyzed. CCL5 (A), CCL2 (B), and IL-8 (C) significantly increased the number of migratory MSCs in a standard Boyden chamber chemotaxis assay. To further characterize the role of CCL5, CCL2, and IL-8 in macrophage-induced MSC migration, neutralizing antibodies targeting these factors were added to macrophage CM prior to migration. While each individual factor induced MSC migration, only CCL5 inhibition reduced MSC migration in response to macrophage CM (D, E, F).

CCL5, CCL2, and IL-8 were present in increased levels in both primary macrophage CM and dU937 CM when compared with control medium. The ability of CCL5, CCL2, and IL-8 to act as a stimulus for MSC migration was tested. The addition of 10 ng/mL recombinant CCL5 induced a 39% increase in MSC chemotaxis compared to control medium (Figure 5A), while 5 ng/mL CCL2 recombinant protein stimulated a 36% increase in MSC chemotaxis (Figure 5B). The addition of 10 ng/mL IL-8 increased MSC chemotaxis from 69.7+/−10.4 to 118.5+/−10.6 MSCs, a 1.7-fold increase (Figure 5C). While CCL5, CCL2, and IL-8 all induce MSC migration, the addition of any of these factors alone does not replicate the 5- to 9-fold increase in MSC chemotaxis seen in response to dU937 CM (604+/− 47.7 migrating MSCs).

We then investigated whether CCL5, CCL2, or IL-8 were required for the induction of MSC migration. A neutralizing antibody directed against CCL-5 attenuated, but did not completely block the response of MSCs to macrophage CM. The addition of 10 µg/mL anti-CCL5 reduced MSC chemotaxis by 27% (Figure 5D), while 10 µg/mL anti-CCL2 did not significantly reduce chemotaxis (Figure 5E). A neutralizing antibody directed against IL-8 showed a trend toward reduced MSC chemotaxis from 604+/−147.7 MSCs to 374+/−125.9, however this was not statistically significant (Figure 5F).

### Activation of JNK is Required for the Stimulation of MSC Migration by Macrophages

**Figure 6:**
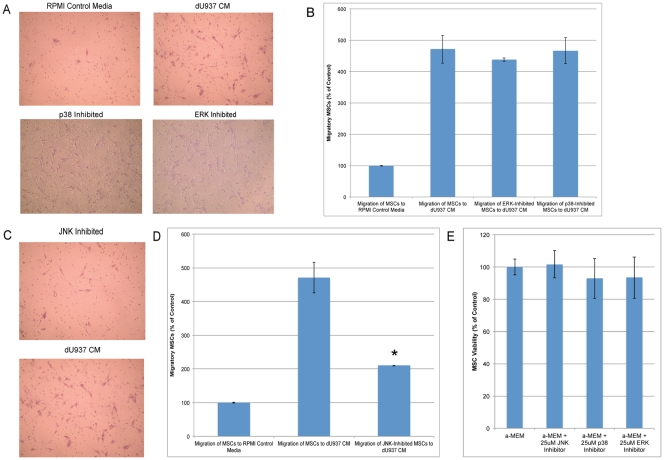
Macrophage-secreted soluble factors induce MSC migration by activating the c-Jun NH2-terminal kinase (JNK) signaling pathway. MSCs were pre-incubated for 1 hour in control medium or control medium containing 25 µM ERK or p38 inhibitor. Neither ERK nor p38 inhibition influenced MSC migration in response to dU937 CM (A, B). MSCs were incubated in 25 µM JNK inhibitor for 1 hour prior to migration assay. JNK inhibition decreased MSC migration by 55% (*p< 0.006, n = 3)(C, D). The MSCs were incubated with the inhibitors for 18 hours after which MSC viability was calculated using trypan blue staining. JNK, p38, and ERK inhibitors did not significantly decrease MSC viability (E).

Because individual external stimuli could not maximally induce MSC migration, we investigated the role of second messenger pathways in this response. P38 MAPK, MAPK/ERK, and SAPK/JNK pathways are involved in chemotaxis in a number of systems [15]–[19]. We characterized the impact of inhibition of these signaling pathways on macrophage-induced MSC chemotaxis. Increased migration of MSCs was seen in response to macrophage CM compared to control medium (2,216+/−212, n = 3) and (471+/−6, n = 3), respectively. Chemotaxis was unaffected by ERK or p38 inhibition (Figure 6A and 6B). However, JNK inhibition significantly reduced the migration of MSCs in response to dU937 CM to 987 (+/−3.5, n = 3), a 55% decrease (Figure 6C and 6D). Trypan blue staining verified that the effect on cell migration was not due to changes in cell viability (Figure 6E). None of the inhibitors used significantly decreased MSC viability at 25 µM.

### Macrophage-secreted Soluble Factors Induce Phosphorylation of JNK1/2/3 and c-Jun

**Figure 7:**
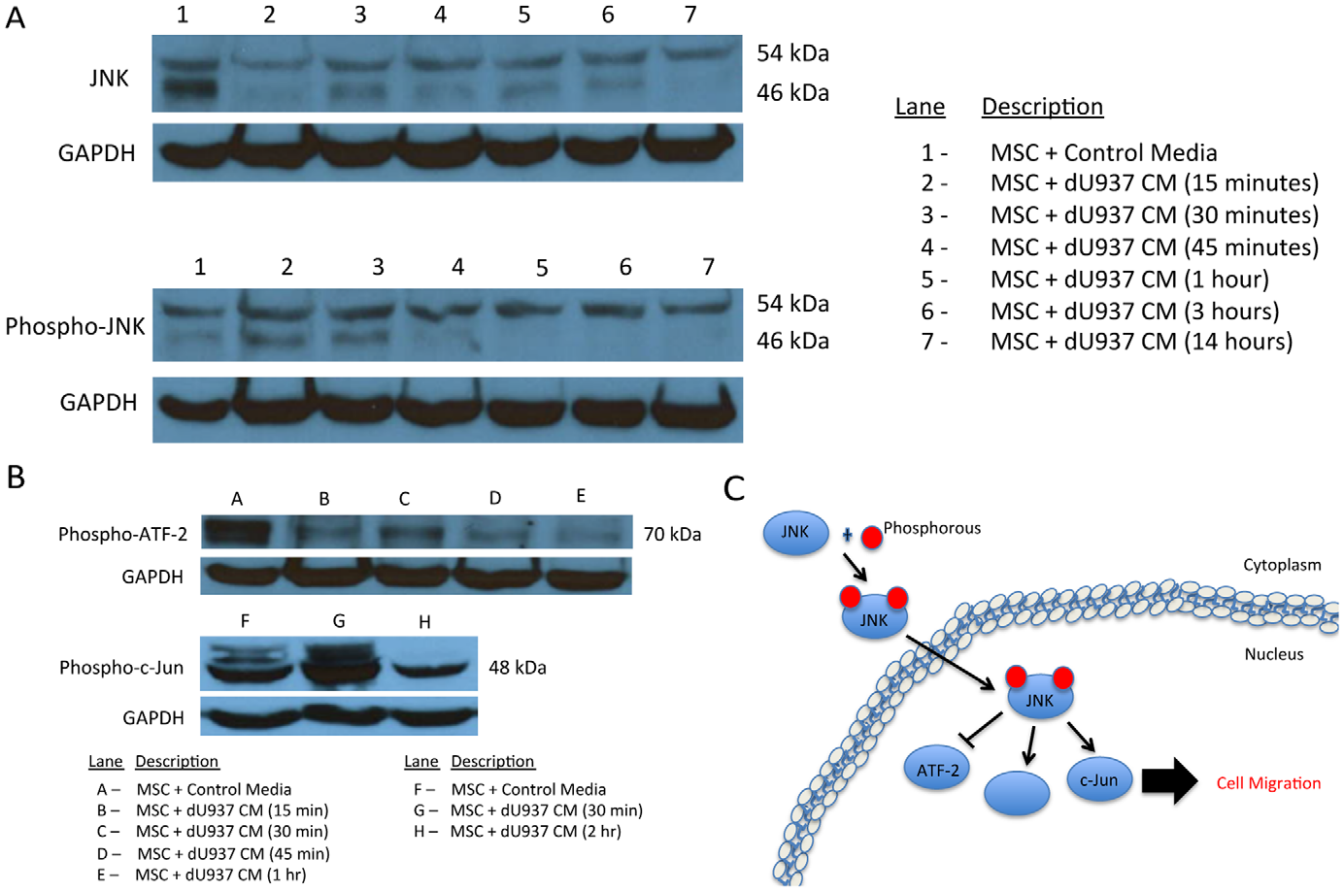
Macrophages induce activation of the c-Jun NH2-terminal kinase pathway in MSCs. To validate the macrophage-induced activation of the c-Jun NH2-terminal kinase (JNK) signaling pathway in MSCs, protein levels of phosphorylated downstream components were assessed by immunoblotting. Exposure to CM from dU937 macrophages induced activation of JNK1, 2, 3 after 15 minutes (A). An increase in the expression of phospho-c-Jun and decrease in the expression of ATF-2 was observed after a 30-minute treatment with dU937 CM (B). These results suggest that in addition to its control of cellular differentiation, proliferation, and apoptosis, the AP-1 transcription factors may also play a role in cellular chemotaxis (C).

In order to verify that JNK was activated in MSCs treated with dU937 CM we determined the levels of phosphorylated-JNK1/2/3 and downstream targets. Both phosphorylated-JNK1/2/3 (Figure 7A) and phosphorylated-c-Jun (Figure 7B) were increased after 30 minutes and returned to control levels by 2 hours. Phosphorylated-ATF-2 levels were not increased at 30 minutes, but rather decreased and remained reduced after 1 hour (Figure 7B) suggesting that MSC migration in response to macrophages may be mediated through c-Jun.

## Discussion

MSCs were first isolated as adherent cells cultured from bone marrow and are non-phagocytic, exhibit a fibroblast-like phenotype, and are able to differentiate into adipose, tendon, muscle, bone, and cartilage *in vitro* [20]. They can be activated and mobilized under the appropriate conditions and localize to solid tumors and injury sites [21]–[29]. Within the injury microenvironment, MSCs differentiate to a myofibroblast phenotype and participate in important aspects of wound healing including regulation of the immune response, inhibition of scarring and fibrosis, angiogenesis, and enhancement of mitosis of stem and progenitor cells during tissue repair [30]–[32]. Additionally, a growing body of evidence suggests that carcinoma-associated fibroblasts (CAF) are derived from MSCs [33]. MSCs differentiate into a CAF phenotype within the tumor microenvironment and support solid tumor growth and metastasis [33], [34].

While the ability of MSCs to localize to sites of tissue damage and adopt specific phenotypes based on microenvironmental cues has been well established, the mechanisms responsible for these aspects of MSC biology are not well characterized. Macrophages are a major component of the inflammatory response in both injury and neoplasm, two cases in which MSC localization and integration is important.

In this work we show that macrophage-secreted soluble factors alter the gene expression and cytokine secretion profiles of MSCs. Macrophages also induce MSC migration and influence the polarization of MSCs to a motile phenotype with increased secretion of IL-6 and IP-10.

Previous work has demonstrated functional interaction between MSCs and macrophages. Macrophages were initially broadly divided into M1 (classically activated macrophages) and M2 (alternatively activated macrophages) subtypes with subsequent expansion into a wide range of different phenotypes that are dictated by the local environment [35]. Co-culture of human monocyte-derived macrophages with MSCs led to increased cell surface CD206 expression and changes in cytokine production by the macrophages in response to stimuli including LPS, interferon gamma, and ionomycin and PMA. Mesenchymal stem cell educated macrophages had increased levels of IL-10 and IL-6 production and decreased levels of IL-12 and TNF-alpha in response to stimuli [36]. This constellation of markers suggests that MSCs induce a novel macrophage phenotype with characteristics of alternatively activated macrophages. In a murine model, bone marrow-derived MSCs suppressed the production of TNF-alpha, IL-6, and interferon gamma and increased production of IL-10 by LPS-stimulated peritoneal macrophages [37], again corresponding to a subtype of macrophages within the M2 spectrum [35]. It is possible that these differences in the response of macrophages to MSCs under various experimental conditions mimic the spectrum of macrophage response in diverse microenvironments *in vivo*. Thus there is accumulating data demonstrating changes in macrophage phenotype in response to MSCs. We present work that focuses on changes in MSC phenotype in response to macrophages.

Two distinct MSC populations, MSC1 and MSC2, have been described and are induced by activation of toll-like receptor (TLR)-4 and TLR-3, respectively [38]. The priming of human MSCs with the TLR-4 agonist lipopolysaccharide (LPS) leads to a pro-inflammatory phenotype with increased secretion of IL-6 and IL-8 that was not able to suppress T-cell activation in culture. Stimulation of TLR-3 with poly(I:C; double-stranded RNA) led to elevated secretion of CCL5 and CXCL10 (interferon-gamma inducible protein-10) by MSCs with less significant increases in IL-6 and IL-8 production. MSCs stimulated with poly(I:C; double stranded RNA) also inhibited T-cell activation in culture. MSC1 and MSC2 polarizations are likely two cellular profiles among an array of *in vivo* possibilities. Macrophage-associated MSCs assume a unique phenotype with some characteristics that more closely resemble MSC2 cells with increased CXCL10 and CCL5 secretion. However IL-6 secretion was also elevated 56-fold over control levels. Messenger RNA levels were increased for CCL7, IL-8, and CCL2 in macrophage-associated MSCs, however they were not secreted at increased levels.

In addition to changes in the expression and secretion of soluble signaling molecules, MSCs also exhibited increased mobility in response to macrophage-derived factors. A number of cytokines and chemokines induce MSC migration [39] and signals for MSC migration are likely to vary depending on local conditions. Recent evidence demonstrates that Akt, ERK, and p38 signaling pathways may be involved in CXCL12-mediated MSC migration. The ERK and p38 signaling pathways have been implicated in the mobilization of MSCs in response to an array of factors including CXCL12 and hepatocyte growth factor[40]–[43]. The JNK signaling pathway has been shown to be involved in the migration of astrocytes [44], tumor cells [45], smooth muscle cells [46], [47], neutrophils [48] and recently in MSC chemotaxis [49], [50]. Our work demonstrates that macrophages induce MSC migration through the production of a constellation of soluble factors. Individually, CCL2, CCL5, and IL-8 stimulate MSC migration. This system has significant redundancy, as no single factor tested could induce maximal MSC migration. However, a common molecular thread for the interaction between macrophages and MSCs appears to be activation of JNK. Small molecule inhibitors targeting the ERK, p38, and JNK transcription factors were used to determine specific pathways involved in MSC chemotaxis. While the individual blockade of several cytokine receptors failed to significantly impair MSC migration, inhibition of JNK activation drastically reduced the response of MSCs to macrophages. This is distinct from other studies of MSC migration, which have focused on signaling through the ERK, p38 and Akt pathways [51]. Based on these results we suggest that targeting JNK in MSCs may represent a unique avenue to manipulating their response to the inflammatory microenvironment. Studies to characterize JNK-induced MSC chemotaxis to other stimuli and the investigation of JNK-inhibited MSC localization to injury models *in vivo* are currently underway.

Our data suggest that macrophages induce a migratory MSC phenotype with increased secretion of CXCL10, CCL5, and IL-6. While this work provides a framework for understanding the impact of macrophages on MSC phenotype, other classes of macrophages may have differential effects on MSC biology. Further exploration of the interaction between macrophages and MSCs in different microenvironments will lead to a more complete understanding of the mechanisms of MSC response during processes such as tissue repair and tumor growth and will provide the framework for targeted gene and drug delivery, tissue regeneration, and novel targets for promotion or inhibition of MSC chemotaxis.

## Materials and Methods

### Primary Culture Human Macrophages

Buffy coats were prepared by the New Brunswick Affiliated Hospitals Blood Center (UMDNJ, New Brunswick, NJ) from peripheral blood samples donated by healthy volunteers. Each sample contained an average of 50 mL of blood. Mononuclear cells were isolated using a BD CPT Vacutainer tubes (8 mL per tube) (BD, Franklin Lakes, NJ). The resulting mononuclear cells were re-suspended in Roswell Park Memorial Institute (RPMI) medium containing 10% fetal bovine serum (FBS)(F0926 - Sigma Aldrich, St. Louis, MO) and 1% penicillin-streptomycin. Cell density was measured using an automated cell counter and cells were plated at a density of 8.0×10^5^ cells/cm^2^. Cells were allowed to adhere overnight and the medium was changed the following day. The cells were incubated at 37°C/5% CO_2_ for 7 days to allow for differentiation of monocytes into macrophages, receiving fresh medium every 3 days. Differentiated monocyte-derived macrophages exhibited increased production of IL-6, IL-8, and TNF-alpha when treated with lipopolysaccharide (LPS) (Figure S1A). In addition, they express low levels of CD204, which is increased by treatment with IL-4 and IL-13 (Figure S1B).

### Primary Culture Mesenchymal Stem Cells

Human bone marrow-derived mesenchymal stem cells (MSCs) harvested from pooled normal human donors were purchased from Lonza (Lonza, Walkersville, MD). Cellular identity was verified by flow cytometry and as well as the ability to differentiate into osteogenic, chondrogenic and adipogenic lineages as previously described [52]. Cells expressed CD105, CD166, CD29, and CD44 and were negative for CD14, CD34 and CD45. MSCs from two separate lots were used in this study. MSCs were expanded in Mesenchymal Stem Cell Growth Medium (Lonza, Walkersville, MD). For experiments using MSCs, cells were cultured in MEM-alpha medium (Invitrogen, Carlsbad, CA) containing 10% FBS and 1% penicillin-streptomycin. Fresh medium was added every 3–4 days and cultures were split to a lower density, using 1:1 trypsin-EDTA 0.25% (2.5g/L trypsin and 0.2 g/L EDTA 4Na in Hanks’ Balanced Salt Solution)(Sigma-Aldrich, St. Louis, MO):phosphate-buffered saline (PBS), once they achieved 80% confluence. All experiments were conducted with passage 3 cells.

### Differentiation of U937 Cells to dU937 Macrophages

U937 cells (ATCC, Manassas, VA) were plated at the desired density in RPMI medium containing 10% FBS, 1% penicillin-streptomycin, and 20 nM phorbol-myristate acetate (PMA) (Sigma-Aldrich, St. Louis, MO). Cells were differentiated for 4 days and then used for experiments.

### Immunoblotting

Cells were trypsinized to remove adherent cells from the tissue culture flask. The cell pellets were washed with PBS and re-suspended in 150 µL 1x radioimmunoprecipitation assay (RIPA) buffer with protease inhibitors. Each sample was sonicated for 10–15 seconds to shear the DNA and reduce sample viscosity. The samples were cooled on ice for 30 minutes, and centrifuged at 14,000 RPM for 20 minutes at 4°C. A Bradford Assay determined protein concentration and lysates were separated on an 8% SDS-polyacrylamide gel. Proteins were transferred to a nitrocellulose membrane for 1 hour. The nitrocellulose membrane was washed with 1x Tris Buffered Saline (TBS) for 5 minutes followed by incubation in blocking buffer for 1 hour at room temperature. The membrane was washed 3 times for 5 minutes each in TBS/T (1x TBS + 0.1% Tween-20) was buffer. The membrane was incubated in primary antibody (at the appropriate dilution) overnight at 4°C with gentle agitation. The membrane was washed 3 times for 5 minutes each in TBS/T. The membrane was incubated with HRP-conjugated secondary antibody (1∶2,000) and HRP-conjugated anti-biotin antibody (1∶1,000) (Cell Signaling Technology, Danvers, MA) in blocking buffer for 1 hour at room temperature with gentle agitation. Again, the membrane was washed 3 times for 5 minutes each in TBS/T. The membrane was incubated in Pierce ECL western blotting substrate (Thermo Scientific, Rockford, IL) with gentle agitation for 1 minute at room temperature. Excess developing solution was drained, the membrane was wrapped in plastic wrap and exposed to x-ray film.

### Conditioned Medium (CM) Preparation

We define conditioned medium as post-culture supernatant without any additional supplements. For the generation of macrophage CM, either monocyte-derived macrophages or differentiated U-937 cells were cultured at 80% confluence in RPMI medium with 10% FBS. The medium was changed, again using RPMI+10% FBS. After 24 hours of incubation, medium was collected from the cell culture and centrifuged for 5 minutes at 1200 rpm. After centrifugation, the CM was filtered using a 0.45 µm pore size vacuum-assisted filter unit (Millipore, Temecula, CA). In all experiments where CM was used in further cell culture, CM was used immediately after being harvested.

### Cytokine ELISA

Conditioned medium was harvested from the cell culture and filtered through a cellulose acetate membrane with 0.45 µm pore size (Corning, New York, NY). Harvested CM was stored at −80°C overnight prior to analysis. All samples, including controls, contained FBS. The cytokine profile of each sample was analyzed using the Bio-Plex suspension array system (Bio-Rad Laboratories, Hercules, CA). The beads were mixed with the samples and incubated to react with the specific analytes. The addition of an analyte-specific biotinylated detection antibody and a fluorescently labeled reporter molecule, streptavidin-PE, completed the sandwich immunoassay. The Bio-Plex array reader aligned the beads single file through a flow cell where two lasers individually excited the beads. Digital signal processors and the Bio-Plex Manager software recorded the fluorescent signals and quantitated the amount of analyte captured in each sample. All samples were assayed in triplicate. Conditioned medium samples displayed in Figure 2 were analyzed by the Cytokine Core Laboratory at the University of Maryland (Baltimore, MD).

### Transwell Migration Assay

The assay was run as previously described [53]. A migratory stimulus (cells or CM) was plated into the well of a 24-well notched tissue culture plate (BD Falcon, San Jose, CA) in a total volume of 700 µL of culture medium. For primary macrophages, the cells were isolated from human peripheral blood samples (as described above) and plated directly into the 24-well plate and allowed to differentiate for 7–10 days prior to performing the experiment. For dU937 cells, U937 cells were plated in 20 nM phorbol 12-myristate 13-acetate (PMA) directly in the 24-well notched plate and allowed to differentiate for 4 days before performing the migration assay. When CM medium was used as a migratory stimulus, 700 µL was added into the 24-well notched plate and the migration assay was run immediately. Control base medium was RPMI+10% FBS, as used to culture macrophages. Once the stimulus was prepared, cell culture inserts (BD Falcon, San Jose, CA), containing an 8 µm pore size uncoated polyethylene membrane, were added to each well. 7.5×10^3^ human MSCs were added to the upper chamber of the cell culture insert in 500 µL of minimum essential medium (MEM)-α supplemented with 10% FBS and 1% penicillin-streptomycin. The tissue culture plates were then incubated at 37°C/5% CO_2_ for 18 hours. MSCs were allowed to migrate through the membrane towards the stimulus during this incubation. After 18 hours, the assay was terminated by aspirating the medium out of the top and bottom chambers, washing the insert with PBS, removing non-migrated cells out of the top chamber using a cotton swab, and fixing and staining the cells in the membrane with crystal violet. The number of MSCs that migrated was quantified manually using an inverted microscope.

The human c-Jun N-terminal kinase (JNK) 1, 2, and 3 inhibitor, SP 600125 (Tocris Bioscience, Ellisville, MO), p38/MAPK inhibitor, SB 203580 (Tocris Bioscience, Ellisville, MO) and MAPK/ERK inhibitor, PD 98059 (Millipore, Temecula, CA) were used to analyze the role of these signaling systems in MSC migration. Small molecule inhibitors were added at a concentration of 25 µM to the MSC culture medium for 1 hour prior to MSC harvesting and transwell migration study. In addition, inhibitors were added with the MSCs to the upper chamber of the transwell migration assay at the same concentration.

### RT-PCR

RNA was isolated from cell cultures using the Qiagen RNeasy Mini Kit (Qiagen, Valencia, CA). RNA quality was assessed by UV spectrophotometry, checking the RNA concentration and purity. cDNA was synthesized using the RT2 First Strand Kit (SABiosciences, Frederick, MD). Pathway-focused gene expression analyses were conducted on the samples using RT2 Profiler PCR Arrays (SABiosciences, Frederick, MD). The Human Inflammatory Cytokines and Receptors PCR array (PAHS-011 - SABiosciences) profiled the expression of 84 key genes involved in mediating immune cascade reactions during inflammation. Data was analyzed with the SABiosciences RT2 Profiler Web-Based PCR Array Data Analysis software, which automatically performs the ΔΔCt fold-change calculations from the uploaded raw threshold cycle data.

### Cell Viability

To assess cell viability a Vi-Cell cell viability analyzer (Beckman Coulter) was used for cell counting. To test the viability of MSCs after macrophage association, MSCs cultured in macrophage CM were compared with MSCs cultured in control media (Figure S2).

### Statistical Analysis

Student’s *t*-test was performed to compare results. A *p* value <0.05 was considered statistically significant. Data are presented as mean ± standard deviation.

## Supporting Information

Figure S1 **Macrophages respond to activating stimuli by increasing secretion of soluble factors and increasing expression of appropriate markers.** Lipopolysaccharide (LPS) stimulation of the macrophage population induced increased secretion of multiple soluble factors including IL-6, TNF-α, CCL3, CCL5, and CXCL12 (A). Activation of macrophages with IL-4 and IL-13 increased the level of CD204 expression (B). These results suggest that the macrophages used in this study responded appropriately, as described in the literature, to multiple activation factors.
(TIF)

Figure S2 **Cellular viability of MSCs is unaffected by stimulation with macrophage conditioned medium.** Culturing of MSCs in dU937 conditioned medium did not alter cell viability when compared with MSCs cultured in control medium (RPMI supplemented with 10% FBS).
(TIF)
