# Supplementary figures and images for: Macrophage-Associated Mesenchymal Stem Cells Assume an Activated, Migratory, Pro-Inflammatory Phenotype with Increased IL-6 and CXCL10 Secretion
Source: PLoS One. 2012 Apr 4;7(4):e35036. doi: 10.1371/journal.pone.0035036 (PMC3319627; doi:10.1371/journal.pone.0035036)

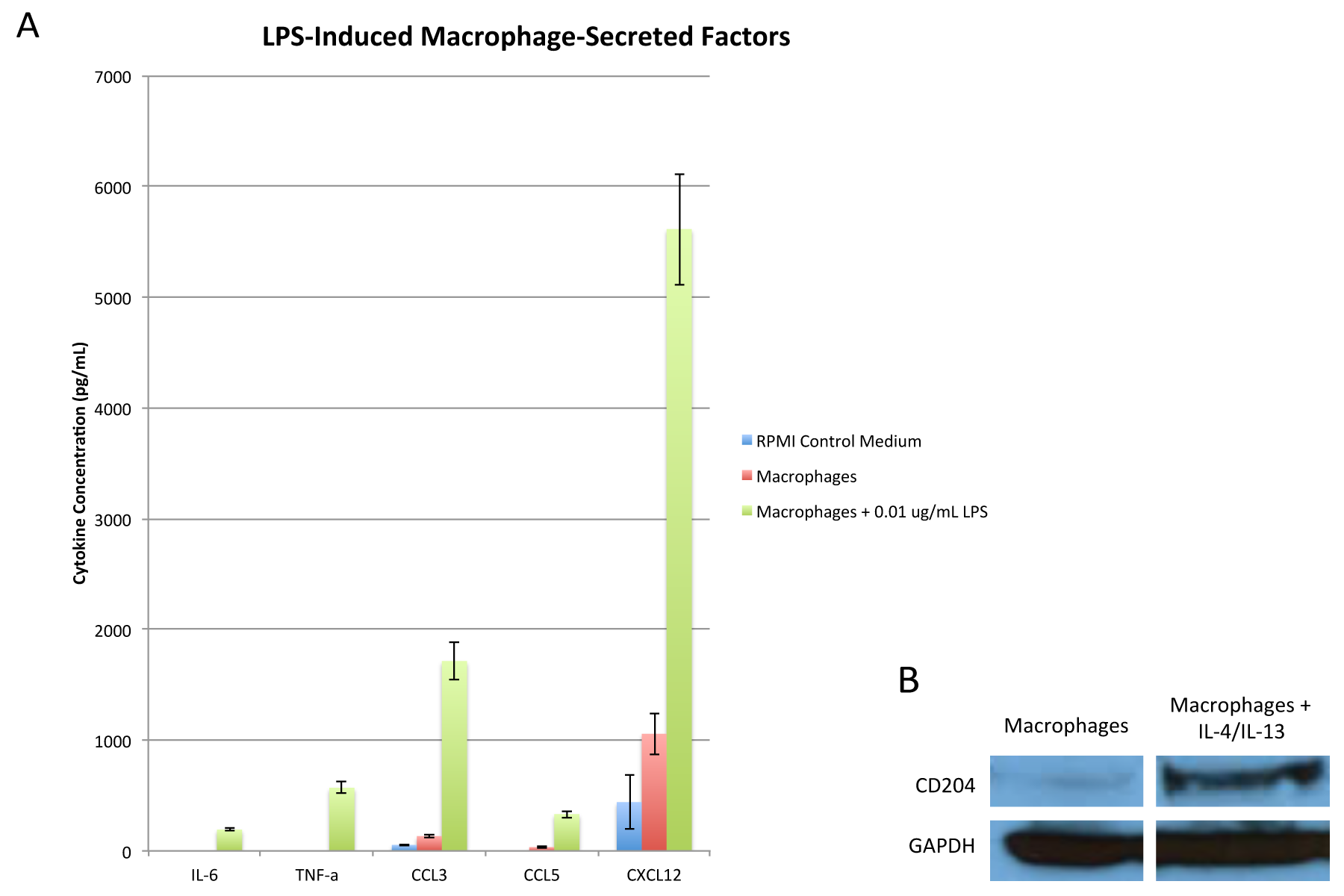

Supplement: Figure S1 — Macrophages respond to activating stimuli by increasing secretion of soluble factors and increasing expression of appropriate markers. Lipopolysaccharide (LPS) stimulation of the macrophage population induced increased secretion of multiple soluble factors including IL-6, TNF-α, CCL3, CCL5, and CXCL12 (A). Activation of macrophages with IL-4 and IL-13 increased the level of CD204 expression (B). These results suggest that the macrophages used in this study responded appropriately, as described in the literature, to multiple activation factors. (TIF) [file pone.0035036.s001.tif]

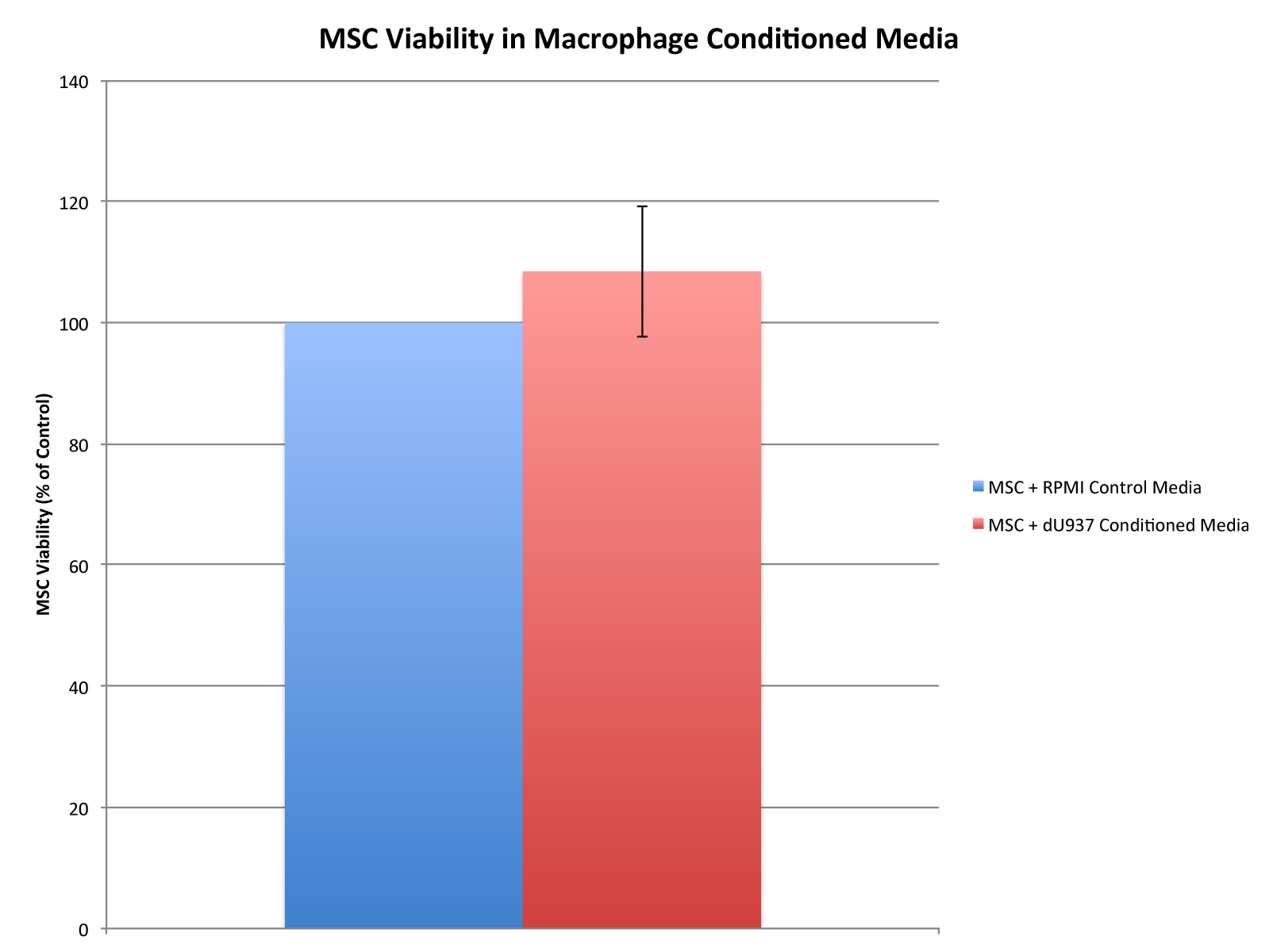

Supplement: Figure S2 — Cellular viability of MSCs is unaffected by stimulation with macrophage conditioned medium. Culturing of MSCs in dU937 conditioned medium did not alter cell viability when compared with MSCs cultured in control medium (RPMI supplemented with 10% FBS). (TIF) [file pone.0035036.s002.tif]
